# Indents

**Published:** 1922-01

**Authors:** 


### INDENTS

**☞Brief Articles of Technical or Scientific Value will receive notice and comment. Contributions invited.**

---

Dental Fatigue. It is now known that shock is exhaustion of the medullary centers, the result of irritating the sensory nerves. This condition, in a milder degree, constitutes fatigue. Shock is dependent upon to sudden, too frequent, too painful, too forcible and too prolonged stimulation of the afferent nerves. Dental shock is caused in the same way, and is just as harmful and dangerous as surgical shocks. Dental fatigue bordering on shock should be handled with great care, and patient should not be subjected to prolonged or excruciating pain.—W. H. De Ford, *Digest.*

---

Haemostatics. As a haemostatic, turpentine, in my opinion, is superior to anything else in dental cases. I have had cases where Monsel's salt, adrenalin and other preparations failed. Put in a plug of cotton wool saturated with turpentine, and bleeding will cease, and, furthermore, the socket will heal in a clean manner, a characteristic not possessed by any other drug.—Fred C. Deakins, *Moree* (*Dental Science.*)

---

A Relief for Bony Vaults in Full Upper Dentures. It is a matter of common observation that many dentures lose their suction after they have been worn for a short time. Many claim that this is due to the denture impringing too severely on the bony ridge in the vault of the mouth. The old-time large suction chamber, though designed to create suction, really acted as a relief for these hard bony ridges.

All operators are agreed that a relief must be provided for these bony prominences if the denture is to prove successful; but there is no unanimity regarding the proper method to be used in accomplishing this. Some take the position that the relief should be provided for on the model; others provide for it on the completed denture.

In *The Pacific Dental Gazette*, Dr. A. Lockwood takes up this subject and suggests a remedy that is reliable and does away with all guesswork. First, before taking the impressions, carefully examine the mouth with your finger; determine the extent and shape of the bony ridge. Now take some tinfoil and cut to approximate the bony part. Place it in the mouth and burnish it over the part. Keep trimming it until you are certain by digital manipulation and feeling that it covers all the bony part and no more. Now, remove it and fold a piece of tin-foil a sufficient number of times to make it the required thickness of the relief. You can determine this by the yielding of the surrounding soft tissues. The softer the tissues the greater should be the relief. When you have secured tin-foil of the required thickness, place the pattern of tin-foil that you have previously fitted over the bony part and cut a new piece exactly to this pattern. Now dry the vault over the bony part, wet the tin-foil and burnish to place. Better burnish the relief first, to be sure you have it where it is needed; then wet and apply powdered gum tragacanth to the palatal side of the tin-foil and stick it into place. Now take the impression. If using the Greene system, the tin-foil will stay in the impression through all the subsequent manipulations to obtain muscle trimming, etc. When the impression is ready to cast, first remove the tin-foil. The model will then have suitable relief sections to take care of the bony portions in the palatal vault.

---

Acid Mouth Washes. Ever since Miller and others have advanced the theory that lactic acid resulting from food fermentation is the causative factor in the production of caries, it has been the ambition of the dental profession to neutralize the acid, in the belief that if this is successfully done decay will be eliminated. The materials which obviously suggested themselves were milk of magnesia, lime water and similar alkalies. The manufactures of tooth pastes and mouth washes acting upon information obtained from dentists and dental literature have concentrated their efforts on "antacid" preparations and with but few exceptions that is the principal merit which they claim for their products.

Recent investigations, however, seem to indicate that we shall have to revise our opinions on the subject. In advocating acid neutralizing agents as decay preventatives we have overlooked the plaques. It is not the free lactic coming in contact with the teeth which does the damage, but the acid remaining undisturbed beneath the mucous plaques and this can not be reached by any of the alkalies, because when an alkaline solution is applied to mucine the result is a viscid mass, which can not be easily disengaged from its close contact with the surface because of its adhesiveness; even the tooth brush will fail to remove it. The only effective way of dislodging the mucine plaques from the teeth is by the use of an acid, because acid curdles the mucine and makes it lose its grip, so that it can be easily flushed away.

This idea is not altogether new; many of us are familiar with the work and writings of Dr. Pikrell and others; the only reason in our opinion why the acid mouth washes have not been more generally adopted is for fear that they may be injurious to the enamel. Such fear, however, is groundless; free acid in the mouth does absolutely no harm. The best proof is the fact that many of us eat grape fruit or oranges every day for several months in the year without any deleterious effect on our teeth.

A ten per cent solution of fruit vinegar swished through the mouth for about a minute, then followed by a copious washing with plain water is without question the best mouth wash that we can recommend to our patient.—*Dental Facts.*

---

THE IMPORTANCE OF THE LOWER THIRD MOLAR. There have been many students of the masticating apparatus of the human being, but as far as I have been able to find, none of them have dwelt upon the importance of the third molar. No investigator, as far as I have been able to find, has given the matter consideration.

We will take up the functions of the third molars as I see them, the first of which is grinding. This we will admit without further argument. The second function is the guiding of the mandible from lateral occlusion to central occlusion. The third is the guiding of the mandible from protrusion to normal occlusion. And the fourth function which works in harmony with the former three functions, is the protection of the tissues surrounding the condyle.

Former students of the subject have looked to the condyle and the condyle tract for guidance in restoring the grinding apparatus. The importance of the third molars becomes apparent upon the study of the position of the third molar. As a start we will assume that the axis of the first molar is vertical. The second molar, possibly in some cases 20 degrees from the vertical, while the third molar varies in a much greater extent; being some times as much as 45 degrees from the vertical. The importance of this variation becomes apparent to the student of the subject who is studying the subject from the position of cusp control.

It will be seen that, if the first molar presents cusp of 45 degrees standing vertical, that the second molar while its cusp presents 45 degrees when we stop and consider the axis of the second molar, we find sharper cusps than 45 degrees as working surfaces, in fact almost to the extent of 60 degrees from the vertical. A still greater variation is found in the third molar, and if it varies from the vertical to the extent of 45 degrees, and its cusps are 45 degrees, much to our surprise we find that the working angle of the cusps is nearly 90 degrees. The importance of this will be made plain to us if we will stop and consider the fact of mastication. To perform this function the mouth is opened, food is passed between the lips and the front teeth, and the food is transferred between the back teeth and the cheek. The movement of the jaw in closing proceeds laterally to the working side, the cusps of the upper and lower teeth meeting in lateral occlusion. The bolus of food holding the teeth apart, prevents the inclined plane of th cusps above, and below from sliding or guiding the jaw into central occlusion, as would be the case if the food was not between the teeth. We must depend upon some other force or guiding power that the inclined cusps of the teeth on the working side.

A study of the subject will make it plain to us that the power to return the jaw to central occlusion, with the bolus of food between the teeth, the jaw being in lateral occlusion on the working side, is presented in the natural subject by the shape of the third molar and its position in the jaw on the opposite side to the working side. While both third molars are of importance, the student of the subject recognizes the fact that the lower third molars is of greater importance than the upper. Upon protrusion of the mandible the lower third molar, in the act of biting, occludes with the upper second molar.

The importance to prothodontist more particularly in his endeavor to restore the full lower portion of the grinding apparatus, of the lower third lower and of making use of three of the four functions, as set forth above, (not using the grinding function) in the construction of lower plates. To accomplish this the outercusps of the third molar above, and the inner cusps of the third molar below are dispensed with, thus still retaining the guiding functions of the third molar upon the opposite side of the jaw to the working side.—Wm. M. Stanbrough, Newburg, N. Y.

---

Capping Exposed Pulps. There is one class of pulp-exposures which frequently presents itself in a busy practice. This class comprises those cases in the mouths of young patients when the pulp is exposed through decay, and the roots of the tooth have not yet been fully developed.

Every effort should be made to cap such a pulp and thereby save it, if only for a year or two. While as a rule it may be expected that the pulp will die, there remains the possibility that it may live to complete the formation of the root. Whenever a capping is undertaken under these conditions a temporary filling should be placed in the cavity and the vitality of the pulp tested at regular stated intervals, so that the pulp may be promptly removed if it dies, and if upon removal of the pulp, it should be found that the apical foramen is so large that it is impossible to make a proper root-filling, the only alternative is to extract the tooth. If the first capping which has been judiciously made fails, the case should be abandoned at once, as every renewed attack of pain marks an extension of the inflammation in such cases; besides the first effort has been made under the very best conditions which could occur in this particular case, and a repetition of the effort is simply to worry the patient, without accomplishing anything. During the period of calcification, the apical end of the partially formed root is wide open so that there is little danger of the death of the pulp from strangulation. There is room for both arteries and veins to become enlarged, during the process of repair to simple injury. Of such pulps, if only slightly exposed while excavating, a considerable number will live if carefully capped, and protected from further irritation, while those exposed by caries will generally die, although a few live.

Another class of cases that present themselves in which it is justifiable to cap the pulp is in those cases of exposure in which it would be difficult to adjust rubber dam, to aseptically remove the pulp and thoroughly fill the canals. Front teeth are excluded on account of the liability of the tooth-structure discoloring after the pulp has been removed.

One difficulty in the treatment of pulp capping is the control of the patient, or in bringing the patient to a realization of the cause of the condition and the treatment necessary to allow the pulp to recover. It is a good rule for the dentist to emphasize to the patient that one of two things will probably occur. Either the pulp will gradually recover, or the pain may become worse and then the tooth may suddenly be free from pain altogether. The patient should be warned that if the latter occurs, the probability is, that the pulp has died, and there is a danger that an abscess will develop unless the pulp is promptly removed. In any case, the dentist should, if possible, have the patient return at regular intervals in order that the progress of the case may be watched.

Indications for capping are:

(1) During the period between the time of the eruption of the tooth and the complete calcification of the root, while the apical foramen is large, whether exposed by caries or in excavating.

(2) Slight exposure with hand excavation in fully-formed teeth.

(3) Never in fully-formed teeth if exposed by caries.

(4) Never if exposure is made with a bar.

*Technique.*—In the effort to save an exposed pulp, the aim should be to avoid as far as possible any further irritation. To which end the following precautions should be observed:

(1) The hyperæmic pulp must be restored to normal before final capping; this can be done by the use of an anodyne.

(2) The dentine overlying the pulp must be thoroughly sterilized, not by the usual method of simply applying a germicidal solution to the cavity for a short time, as this has without doubt been the chief cause of many failures.

(3) Pressure, in applying the material for capping, must be avoided.

Break down all overhanging edges of enamel and remove as much of the débris and softened dentine as can be done without pain or injury to the pulp; flood the cavity with a mild non-irritating, antiseptic solution at body temperature. Absorb excess with cotton and adjust dam. With a sharp spoon excavator remove the remaining carious dentine, unless in so doing a large exposure would be made, in which case it is better to leave the layer over the pulp and depend on the sterilizing agent. The delicate pulp tissue will not tolerate much abuse. The dentine can now be sterilized by sealing in the cavity for a week or two the following germicidal and anodyne remedy: Menthol, gr. xx; Thymol, gr. xl; Phenol, ʒ iii. Under this treatment the pulp will very likely return to normal from its hyperæmic condition. At the next sitting, should the history be favorable during the interval, apply the dam, and remove previous dressing carefully, and gently cover the exposure and dentine immediately over the pulp with a thin paste of pure precipitated calcium phosphate and phenol compound, oil of cloves, or eugenol. Place the paste to one side of the cavity and gently coax over the exposure, in such a manner as to exclude all air. This paste is antiseptic and non-irritating and should cover the entire dentine immediately over the pulp as well as the exposure, thereby protecting the pulp from the irritating properties of the phosphoric acid of the temporary cement used to cover the paste. It is best to fill the cavity now with temporary cement and wait for a few months or perhaps a year before inserting any permanent filling or inlay.—H. S. Abar, D.D.S., L.D.S., *Dominion Dental Journal,* (August, 1921).

---

Dental Ethics. When consulted by the patient of another practitioner, the dentist should guard against inquiries or hints disparaging to the family dentist or calculated to weaken the patient's confidence in him; and if the interest of the patient will not be endangered thereby, the case should be temporarily treated, and referred back to the family dentist. This is probably the most abused section of the Code of Ethics. Only yesterday a dentist came to me and reported that he had been threatened with suit for malpractice because he had capped the pulp of a tooth. Later the pulp had died, considerable pain was felt, and the tooth had to be extracted. The patient, instead of returning to the dentist who had performed the first operation, had gone to another practitioner who had criticized and ridiculed the operation as performed by the first dentist and made it apparent to the patient that he was entitled to damages. Personally, I would rather trust my own dentistry in the hands of a dentist of average ability who earnestly endeavored to do what was right than to one of exceptional ability who would so unjustly condemn the work of another in order that he might gain financially. There are too many such instances, and every effort of the conscientious ethical practitioner should be used to overcome this habit. Be honest. The other fellow may be right; study his view of the question. You may be benefitted thereby.

